# Use of a Macintosh blade in extrahepatic portal vein obstruction with difficult intubation: two case reports

**DOI:** 10.1186/s13256-016-1001-9

**Published:** 2016-09-06

**Authors:** Azho Kezo, Rajendra D. Patel, Shraddha Mathkar, Sonal Butada

**Affiliations:** 1 Department of Anaesthesiology, Seth G.S. Medical College and King Edward VII Memorial Hospital, Dr. E. Borges Road, Parel, Mumbai, Maharashtra 400012 India; 2 House no 21, Near Green Park, Sixth Mile, Dimapur, Nagaland 782062 India

**Keywords:** Case report, Indian subcontinent, South Asian ethnicity, Extrahepatic portal vein obstruction (EHPVO), Difficult airway, Macintosh, Truview EVO2, Airtraq, Miller, Temporomandibular joint (TMJ) ankyloses

## Abstract

**Background:**

We report the management of two patents from the Indian subcontinent with extrahepatic portal vein obstruction presenting with anticipated difficult airway. A Macintosh blade was used to secure the airway after using various instruments designed for difficult airway. To the best of our knowledge, no case has previously been reported in which a Macintosh blade was used successfully in patients with extrahepatic portal vein obstruction with a difficult airway.

**Case presentation:**

Two women (case 1 and case 2) of South Asian ethnicity with extrahepatic portal vein obstruction presented for an elective splenorenal shunt. They both had micrognathia and restricted mouth openings. They had similar airway profiles with mouth openings of just 2 cm, Mallampati class IV, a thyromental distance <4 cm, a hyomental distance <2.5 cm, and a sternomental distance of 10 cm. Awake intubation was attempted in both patients after standard airway preparation in the form of preoperative 4 % lignocaine nebulization and 2 % viscous lignocaine gargle along with an on-table supralaryngeal nerve block using 2 % lignocaine and transtracheal infiltration with 4 % lignocaine.

The patient in case 1 tolerated the procedure well whereas the patient in case 2 had to be given propofol 60 mg. Endotracheal intubation with a 6.5 mm polyvinyl chloride endotracheal tube was attempted using a Truview EVO2, an Airtraq, and a Miller blade no. 3 but was unsuccessful. Finally, a trial intubation was performed successfully with a Macintosh blade with a stubby handle assisted by a Frova Intubating Introducer in case 1 and a gum elastic bougie in case 2.

**Conclusions:**

Although many instruments have been introduced to manage difficult airways, our experience in these cases suggests that the Macintosh blade can be used first when attempting endotracheal intubation before using other instruments. Patients from the Indian subcontinent with extrahepatic portal vein obstruction are often found to have associated temporomandibular joint ankyloses (hence difficult airways). We hypothesize that a difficult intubation should be anticipated in these patients. Such an association has not been made before.

## Background

Difficult airway and techniques to manage them are of utmost importance and great interest to anesthesiologists [1]. Guidelines have been formulated and various instruments have been introduced for difficult intubation [2, 3]. Here, we present two cases where difficult intubation was anticipated, and in which, despite trying various devices to visualize and secure the airway, only the use of a Macintosh blade proved successful. Our experience with these cases also suggests that extrahepatic portal vein obstruction (EHPVO) is a risk factor for difficult airway. Such an association has not previously been reported.

## Case presentation

Two patients with EHPVO presented with micrognathia, restricted mouth openings, and facial asymmetry. They were both women of South Asian ethnicity from the Indian subcontinent, aged 16 years and 18 years, who came for splenorenal shunt surgery to treat longstanding on-and-off hematemesis as a result of portal hypertension and esophageal varices. Hematemesis could not be controlled with regular esophagoduodenoscopy for esophageal variceal band ligation. Hence, our patients were advised surgical intervention.

Both patients presented with similar findings on airway examination (Table 1).

**Table 1:** Airway examination

	Case 1	Case 2
Mouth opening	2 cm	2.5 cm
Mallampatti grading	IV	IV
Dentition	Normal	1 missing upper incisor
Thyromental distance	3 cm	3 cm
Hyomental distance	2.5 cm	2.5 cm
Sternomental distance	10 cm	9.5 cm
Right mandible	9 cm	6 cm
Left mandible	4.5 cm	8.5 cm

### Case 1

**Fig. 1:**
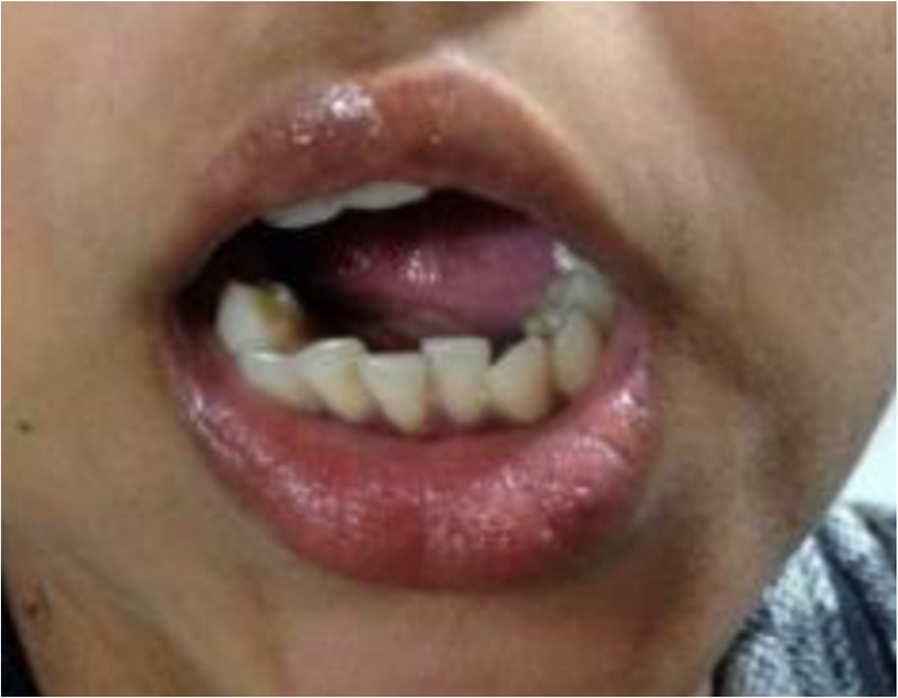
Patient from case 1

With a history of hematemesis on and off for the last 8 years, this was her first time undergoing general anesthesia (Fig. 1).

### Case 2

**Fig. 2:**
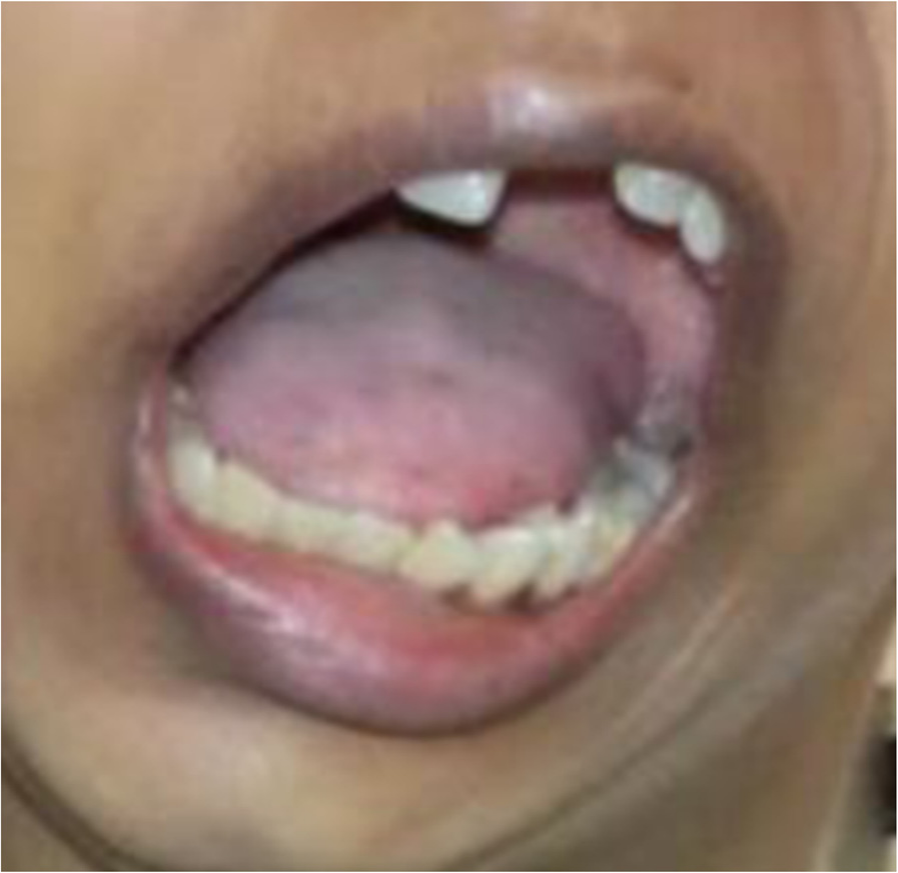
Patient from case 2

Ten years ago, case 2 underwent esophagoduodenoscopy when her hematemesis first started. She was intubated after three attempts using a bougie and Macintosh blade no. 2 owing to her restricted mouth opening and a Cormack-Lehane (CL) grade IV glottic view (Fig. 2). Following this incident, she was advised to undergo temporomandibular joint (TMJ) ankyloses repair, which was done in May 2005 under general anesthesia. Her recovery was uneventful.

### Preoperative preparation

The two patients were prepared similarly for awake intubation, as follows. After a thorough preoperative assessment, the procedure was explained and valid informed consent was taken. Fasting was confirmed and our patient was wheeled into the operating theatre. Standard monitors – pulse oximeter, cardioscope, non-invasive blood pressure monitor, and end tidal gas monitors – were attached and baseline preoperative readings were noted on the table.

We used an 18G intravenous cannula to secure an intravenous line and Ringer’s lactate was started. Piperacillin and tazobactam (2 g and 0.25 g, respectively), ondansetron 4 mg, hydrocortisone 100 mg, and dexamethasone 8 mg were administered intravenously.

A difficult airway trolley was kept ready having Macintosh blades, McCoy blades, Truview EVO2, Miller blade no. 4, Airtraq, stylet, gum elastic bougie, Frova Intubating Introducer, endotracheal tubes (sizes 6 mm, 6.5 mm, 7 mm), laryngeal mask airway, jet ventilation device, emergency cricothyroidotomy set, and a well-functioning suction apparatus.

Because the patients had deranged coagulation profiles [case 1: international normalized ratio (INR) 1.8; case 2: INR 1.8) and low platelet counts (case 1: 45,000/mm^3^; case 2: 40,000/mm^3^), nasal intubation and retrograde intubation were not considered. Platelets (10 mL/kg body weight) and fresh frozen plasma (10 mL/kg body weight) were infused on the morning of surgery under close observation.

### Airway preparation

Viscous gargle with lignocaine 2 % and 4 % lignocaine nebulisation were administered.

A supralaryngeal block with 2 ml of 2 % lignocaine on either side and transtracheal infiltration with 2 ml of 4 % lignocaine and 10 % lignocaine aerosol spray were given. Our patients were then pre-oxygenated for 3 min with 6 L/min of oxygen [1] and the airway was visualized using a Truview EVO2 and Airtraq.

### Case 1

Intubation was attempted with a Truview EVO2, Airtraq, and Miller 3 with the assistance of a Frova Intubating Introducer and a gum elastic bougie but each attempt showed a glottic view of CL grade IV. When a Macintosh blade no. 3 with a stubby handle was used, a CL grade III(b) view was achieved. Her airway was then secured by passing a 6.5 mm endotracheal tube over the Frova Intubating Introducer.

Our patient tolerated the procedure well and her vitals were stable throughout.

### Case 2

Signs of apprehension were displayed by the patient so propofol 60 mg was administered intravenously.

Intubation was attempted using a Truview EVO2 and Airtraq but the glottis view was CL grade IV at each attempt. Finally, when a Macintosh blade no. 3 fixed on a stubby handle was used, the glottic view improved to CL grade III(b). By passing a gum elastic bougie through her trachea and rail-roading a no. 6.5 mm endotracheal tube over it, her airway was secured.

**Fig. 3:**
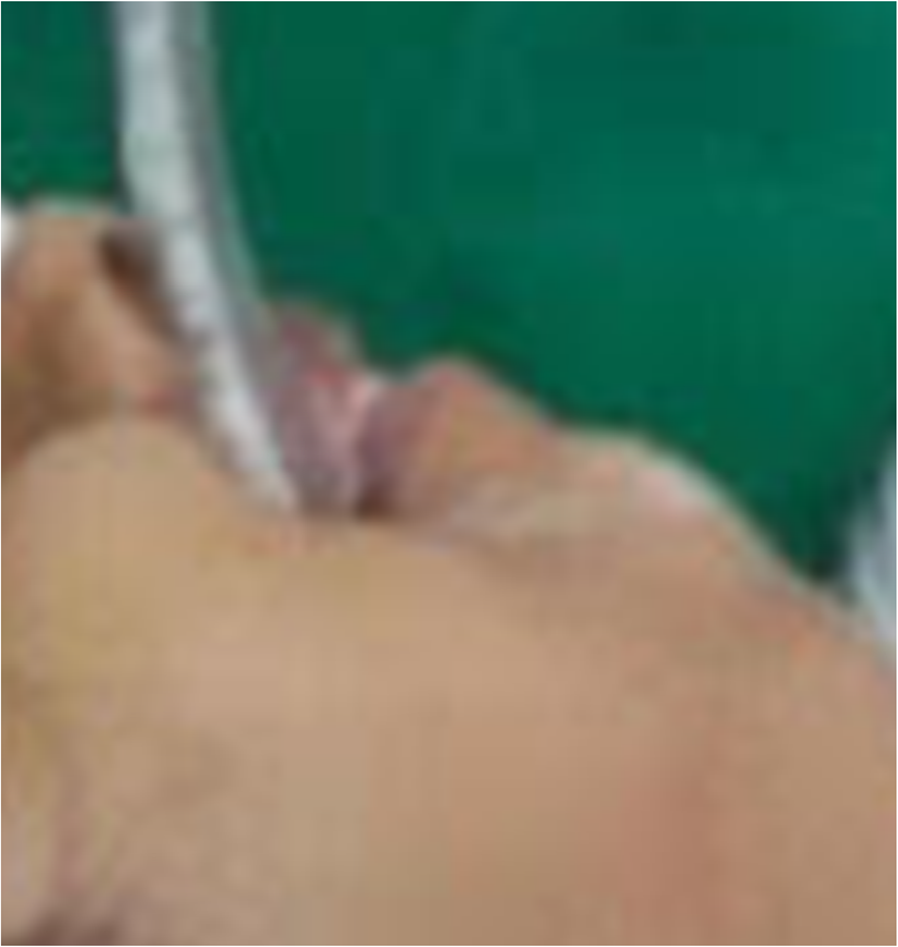
Case 2 after successful endotracheal intubation

Our patient cooperated with the procedure and her vitals were stable throughout (Fig. 3).

## Discussion

Where a difficult intubation is anticipated because of a restricted mouth opening, awake flexible fiber optic intubation (FFI) can be used. It has been considered the gold standard for anticipated difficult intubation [2–4].

Some studies have also suggested that awake intubation using a McGrath video laryngoscope (MVL) is equally successful in anticipated difficult intubation cases. In one case in which the patient could not be intubated by FFI, MVL was instead used successfully [5]. Although MVL has not been found to be faster than FFI, it can be implemented when the user lacks proficiency in FFI. Inexperienced users find awake MVL intubation easier than FFI because FFI has a steep learning curve. MVL can be used as a rescue intubation technique in unanticipated difficult airways [6].

Truview EVO2 has been used for intubations in which the oral, pharyngeal, and tracheal axes are not properly anatomically aligned. The blade has a refraction angle of 46° and has been suggested as a better option when the Macintosh fails to show the glottis opening [7]. In both our cases, a senior consultant (anesthesiologist) tried to navigate the airway using the Truview EVO2 first because the axes of the airway were not aligned. Owing to facial asymmetry, there was narrowing of the airway beyond the tonsils which did not allow enough space for the angled part of the blade to be manipulated.

An Airtraq laryngoscope is a single-use optical laryngoscope that has a guiding channel for inserting an endotracheal tube. The image is reflected onto a proximal viewfinder by a combination of lenses and a prism. It is effective in the intubation of patients with cervical spine immobility and who are morbidly obese [8, 9]. Although its application in malaligned oropharyngeal axes has proved successful, its use in our two cases was futile because the patients had restricted mouth openings.

The Miller blade is a straight blade with a curved tip to improve lifting of the epiglottis. It is designed to facilitate better exposure of the glottis in difficult airways but Amornyotin et al. [10] reported that intubation using a Macintosh blade was more successful than using a Miller blade. The Macintosh blade was used successfully in some cases where endotracheal intubation with Miller blade failed [10].

When the Macintosh blade was used, a CL grade III(b) view of the glottis was achieved. The curvature of the blade is smooth and has a beaked tip. This allowed it to be placed into the vallecula and raise the epiglottis, thereby making it possible for the airway to be secured.

A Frova Intubating Introducer is useful when the glottic view is poor as long as the epiglottis can be visualized. It can be guided into the trachea by gliding it posterior to the epiglottis. Entry into the trachea is confirmed by a grating sensation (tracheal clicks), caused by friction over the tracheal cartilages, and an end tidal carbon dioxide reading on the monitor. Its hollow structure assists in administering 100 % oxygen during the procedure and prevents desaturation.

Because surgery in the two cases was elective, failure to secure the airway despite our best attempts would have prompted a deferral and led to further planning of an awake fiber-optic intubation.

Our institute has observed patients with EHPVO presenting with difficult intubation as a result of TMJ ankyloses [1]. One study suggests that the hypercoagulable state of EHPVO can predispose such patients to TMJ ankyloses [11]. This makes EHPVO a risk factor for difficult airways in patients from the Indian subcontinent.

## Conclusions

The successful use of a Macintosh blade was due to its smooth curvature and beaked tip, which allowed it to be maneuvered through the narrow airway up to the vallecula. Macintosh blades are also frequently used, and so the skills that have been mastered over time may have contributed to its successful use in these cases.

Hence, we emphasize that a Macintosh blade can be the first instrument used in an anticipated difficult airway even if an awake intubation has been planned. Patients will tolerate the procedure with adequate airway preparation using topical anesthesia.

We also hypothesize that EHPVO and TMJ ankyloses are associated, so patients with EHPVO carry the risk of a difficult airway.
